# Bilateral Pulmonary Embolisms: A Hidden Cause Behind a Fall

**DOI:** 10.51894/001c.144435

**Published:** 2025-09-30

**Authors:** Zeelaf Butt

**Affiliations:** 1 Department of Internal Medicine Detroit Medical Center-Sinai Grace Hospital

## 71

### INTRODUCTION

Elderly patients are at high risk for falls. Proper history-taking and workup are necessary to uncover the cause, as differentials in this population are diverse. We present a case of a 75-year-old female with multiple falls, ultimately diagnosed with bilateral pulmonary embolism (PE).

### CASE DESCRIPTION

A 75-year-old female with diabetes and hypertension presented to the ED with profound bilateral lower limb weakness after an unwitnessed mechanical fall. Initial physical exam showed an elderly lady in no acute distress, with clear lungs, unimpressive cardiovascular and GI exams. The neurological exam revealed 4/5 lower limb power without sensory deficits. Reflexes, tone, and bulk were normal. Orthostats, telemetry, and ophthalmological exams were unremarkable.

Labs showed sodium 136, potassium 5.7 (slight hemolysis), an anion gap of 19, beta-hydroxybutyrate 78.7, and glucose 455. UA was positive for glucosuria and ketonuria. CPK was 63, and troponin 6. Trauma workup, including chest, ankle, knee, and pelvis X-rays, CT head, and EKG, showed no acute pathology.

Despite DKA resolution within 24 hours, the patient continued to complain of lower extremity weakness. Her daughter reported increased falls and persistent weakness prior to admission, prompting further workup. A 2D echo showed an EF of 70% with RV systolic/diastolic overload and possible LVOT encroachment. Bubble study confirmed a right-to-left cardiac shunt. TEE revealed a right pulmonary artery thrombus, and CT PE confirmed bilateral proximal PEs with an RV/LV ratio of 2.5, suggesting right heart strain. No DVT was found. Given RV strain and the absence of acute DVT, the PEs were deemed likely chronic. The patient was started on Eliquis, advised to follow up for PFO closure, and discharged to a skilled nursing facility.

### DISCUSSION/CONCLUSION

Given the increased risk of PE in the elderly and its atypical presentations, maintaining a broad differential for unexplained falls or syncope is crucial. A New England Journal of Medicine study found nearly one in six hospitalized syncope cases had an underlying PE. Comprehensive assessment and appropriate imaging are essential for timely diagnosis and treatment, reducing morbidity and mortality.

